# Methylglyoxal Alters the Function and Stability of Critical Components of the Protein Quality Control

**DOI:** 10.1371/journal.pone.0013007

**Published:** 2010-09-24

**Authors:** Carla Figueira Bento, Filipa Marques, Rosa Fernandes, Paulo Pereira

**Affiliations:** 1 Center of Ophthalmology and Vision Sciences, Institute for Biomedical Research in Light and Image (IBILI), Faculty of Medicine, University of Coimbra, Coimbra, Portugal; 2 PhD Programme in Experimental Biology and Biomedicine, Center for Neuroscience and Cell Biology (CNC), University of Coimbra, Coimbra, Portugal; St Georges University of London, United Kingdom

## Abstract

**Background:**

Increased production and accumulation of methylglyoxal (MGO), as well as increased modification of proteins by glycoxidation, are hallmarks of aging and diabetes. MGO was shown to modify proteins and to contribute to the accumulation of damaged proteins that can be toxic to cells. However, the effect of MGO on the cell systems responsible for repairing or degrading damaged proteins is still unclear. In this study, the effect of MGO on the function of the ubiquitin-proteasome system (UPS) and on molecular chaperones, two cooperative mechanisms associated with protein quality control, was investigated.

**Principal Findings:**

In this work it is shown that treatment of cells with MGO leads to accumulation of ubiquitin conjugates and depletion of free ubiquitin. Moreover, MGO significantly decreases the proteolytic activity of the 20S proteasome. Data further shows that MGO decreases the levels of the molecular chaperones Hsc70 and Hsp90 and leads to accumulation of CHIP-, Hsp40- and ubiquitin-containing aggregates. The formation of large aggregates containing CHIP is a consequence of its binding to misfolded proteins and to molecular chaperones. Moreover, dysfunction of the chaperones/CHIP/UPS axis is associated with accumulation of oxidized and argpyrimidine-modified proteins, which is likely to be associated with decreased cell viability. Interestingly, data further shows that MGO-induced stress induces the activation of heat shock factor-1 (Hsf-1), the main transcription factor involved in the regulation of the expression of heat shock proteins (HSPs) and cell response to stress.

**Conclusions:**

The data obtained in this work suggests that MGO impairs both the UPS and the protein quality control dependent on CHIP and molecular chaperones, leading to accumulation of toxic aggregates and increased cell death. However, these MGO-induced changes appear to elicit a response from the Hsf-1 system, which is crucial to help cells to cope with cellular stress and to re-establish homeostasis.

## Introduction

MGO is a highly reactive α-oxoaldehyde that is formed in cells primarily from the triose phosphate intermediates of glycolysis, dyhydroxyacetone phosphate and glyceraldehyde 3-phosphate [1]. This by-product of glycolysis induces rapid and non-enzymatic modification of free amino groups of lysine and arginine residues of proteins, leading to the generation of advanced glycation end products (AGEs) [2]. The posttranslational modification of proteins by MGO is known to contribute to aging, as well as to a number of diseases, including cancer, diabetes and other disorders [1]. Indeed, hyperglycaemia triggers enhanced production of MGO on diabetes [1], [3], leading to vascular dysfunction [4], while AGEs exacerbate and accelerate a number of characteristics of many age-related diseases [5], [6].

MGO is known to induce aggregation and structural modifications in proteins through cross-linking and formation of chemical adducts [7], [8]. In eukaryotic cells, the proteasome contributes to prevent the accumulation of non-functional, potentially toxic proteins. This proteolytic system is of particular importance in protecting cells against harsh conditions, such as heat shock, glycation or oxidative stress [9], and is an important player in the protein quality control system in eukaryotic cells. However, when the generation of obsolete proteins exceeds the capacity of the cell to degrade them, there is a progressive accumulation of damaged proteins, which often become insoluble over time and are potentially toxic to cells [9]. Accumulation of damaged proteins, ultimately, occurs when the proteolytic systems and molecular chaperones, which normally prevent aggregation (for example, Hsc70 and Hsp90) [10], [11], cannot keep up with the rate of production of misfolded or otherwise damaged proteins.

The carboxy terminus of the Hsc70-interacting protein (CHIP) is a key player in protein quality control since it is a molecular link between chaperones and the UPS. Indeed, CHIP has both a tetratricopeptide repeat (TPR) motif and a U-box domain [12]. The TPR motif associates with chaperones, such as Hsp40, Hsc70 and Hsp90, while the U-box domain has ubiquitin ligase activity. Thus, CHIP has a critical role in protein quality control by ubiquitinating misfolded or post-translationally modified proteins through interaction with molecular chaperones [13].

It has been suggested that the activity of the proteasome can be compromised upon aging [14] and diabetes [15]. For example, the levels of the proteasome activator PA28 are decreased in muscle extracts of diabetic rats [16], which might result in accumulation of glycated and toxic proteins, typically observed in diabetic tissues. Furthermore, formation of amyloid-aggregates, which is usually associated with aging and diabetes, is highly enhanced by MGO and other aldehydes [17] and this is known to impair the UPS, leading to apoptosis [18]. On the other hand, it has been suggested that UPS activity decreases with aging, which may further account for the accumulation of modified and toxic proteins in cells, contributing to cell injury and death [14].There is also increasing evidence showing that molecular chaperones, such as α-crystallin and Hsp27 [19], [20], are themselves major targets of MGO-induced modification and aggregation. These and other evidences indicate that age- and diabetes-dependent accumulation of MGO is likely to account for impairment of the protein-quality control mechanism. However, the effect of MGO in the function of UPS and on molecular chaperones is still not clear.

Herein, it is shown that increased levels of MGO lead to accumulation of ubiquitin conjugates and depletion of free ubiquitin in the cell. The accumulation of ubiquitin conjugates is presumably due to both MGO-induced impairment of UPS and MGO-induced disruption of chaperones. Data presented in this work further shows that MGO downregulates Hsc70 and Hsp90 and promotes the formation of large aggregates containing Hsp40, CHIP and ubiquitin. Consistently, increased levels of MGO lead to accumulation of modified proteins and decreased cell viability. Moreover, MGO-induced cell damage elicits a heat-shock response by activation of Hsf-1, presumably as part of a more general strategy of cell response to stress.

## Results

### MGO impairs proteasome activity and leads to accumulation of ubiquitin conjugates

Increased production and accumulation of MGO have been associated with cell and tissue dysfunction on diabetes and aging [1], both characterized by decreased proteasome activity [14], [16]. However, the putative effect of MGO on the proteasome activity and on the accumulation of modified proteins remains to be clarified.

To evaluate the effect of MGO in the proteasome activity, we used a human retinal pigment epithelium cell line (ARPE-19). This cell type represents an appropriate model for this study since it has a very active and functional UPS [21], [22]. Moreover, the retinal pigment epithelium (RPE) has a high rate of glucose consumption and its ubiquitin-proteasome system has been shown to be affected during aging and diabetes [5], [6], [23]. Indeed, formation of AGEs is a trigger for the onset of age-related phenotypes on choroid and RPE cells [6].

**Figure 1:**
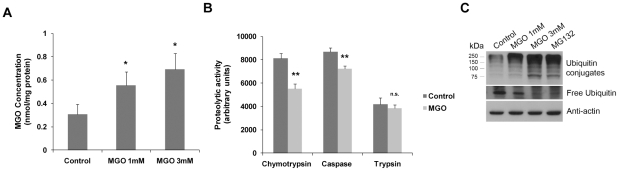
MGO decreases the 20S proteasome activity, depletes free ubiquitin and leads to accumulation of ubiquitin conjugates. (A) ARPE-19 cells were treated with 1 mM or 3 mM of MGO for 3 hours. Cells were then lysed and the intracellular levels of MGO were determined by HPLC after derivatization with DDB. The results represent the mean ± SD of at least three independent experiments. * p<0.05, significantly different from control (one-way ANOVA). (B) ARPE-19 cells were treated with 3 mM MGO for 3 hours and the cell extracts were used to measure the proteolytic activities of the 20S proteasome (chymotrypsin-like, caspase-like and trypsin-like activities) using specific fluorogenic substrates. The results correspond to the measurement at 30 minutes of activity and represent the mean ± SD of at least three independent experiments. ** p<0.01, significantly different from control; paired T test. (C) ARPE-19 cells were treated with 1 mM MGO, 3 mM MGO or 20 µM MG132 for 3 hours and the proteins were separated by SDS-PAGE, transferred to PVDF membranes and probed with monoclonal antibodies against ubiquitin and actin.

Prior to test the proteasome activity, we observed that incubation of RPE cells with 1 mM or 3 mM MGO for 3 hours resulted in an intracellular accumulation of MGO of 0.5±0.12 and 0.7±0.14 nmol MGO/mg protein, respectively, which corresponds to a 1.8 and 2.3 fold increase as compared to control (Figure 1A). Although the high concentrations used in this work are not found in the plasma and tissues, we observed that they induce an intracellular accumulation of MGO that is consistent with the levels found in tissues of diabetic animals [24]. Moreover, the obtained intracellular levels of MGO using these concentrations are similar to those observed in other studies using cell culture systems [25].

To assess the 20S proteasome activity, ARPE-19 cells were treated with MGO and the cell extracts were used to measure the three peptidase activities of the 20S proteasome using fluorogenic substrates. Data shows that these MGO concentrations decrease the chymotrypsin-like and caspase-like activities of the 20S proteasome, by about 32% and 17%, respectively (Figure 1B). Immunoblot analysis of ARPE-19 cell extracts treated with MGO revealed that MGO increases the accumulation of ubiquitin conjugates and decreases the levels of free ubiquitin (Figure 1C). This result is comparable to data obtained in the presence of a proteasome inhibitor (such as MG132) and suggests that MGO impairs proteasome activity.

Considering that the fluorogenic substrate used to assess the chymotrypsin-like activity of the proteasome may also be a substrate for other chymotrypsin-like proteases, such as calpains [26], one may not exclude the role of MGO in the inhibition of other proteases. Moreover, MGO may also exert some effects on the activity of deubiquitinating enzymes, as well as on the activity of ubiquitin-conjugating or ubiquitin-ligase enzymes, inducing changes on the total levels of ubiquitin-conjugates in the cell. However, the fact that MG132 and epoxomicin inhibited by over 90% the degradation of fluorogenic substrates, specifically Suc-LLVY-AMC, (data not shown) strongly suggests that the major contribution for the degradation of these substrates occurs indeed *via* proteasomal degradation.

### MGO decreases the levels of Hsc70 and Hsp90

**Figure 2:**
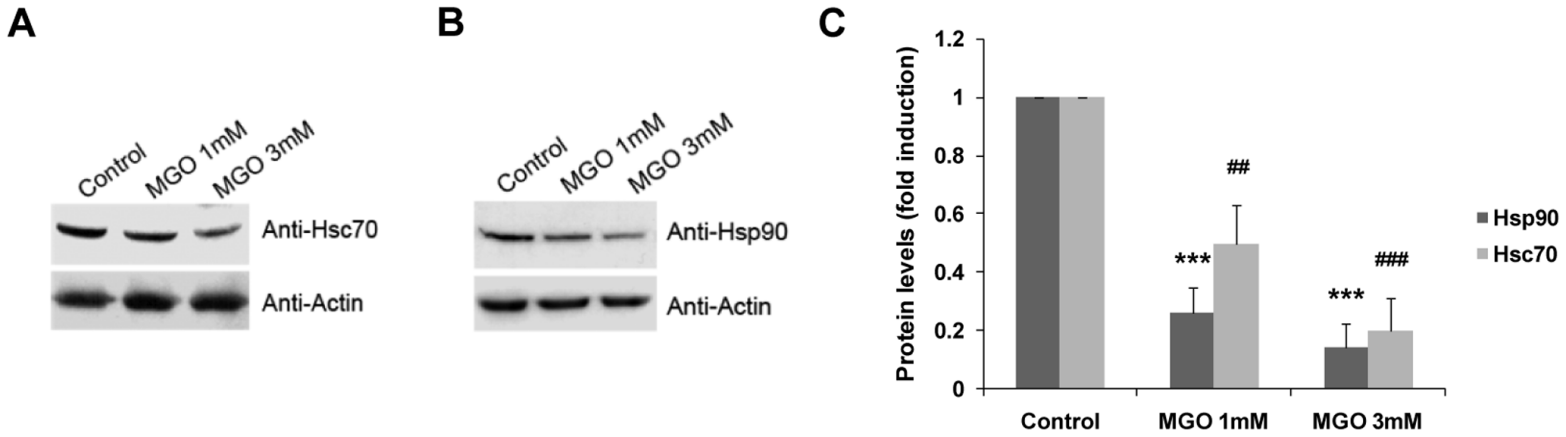
MGO decreases the levels of Hsc70 and Hsp90. (A, B and C) ARPE-19 cells were treated with 1 mM MGO or 3 mM MGO for 3 hours and the cell lysates were analyzed by western blotting using anti-Hsc70 (A), anti-Hsp90 (B) and anti-actin antibodies. The quantified results represent the mean ± SD of at least three independent experiments. *** p<0.001, ^##^ p<0.01, ^###^ p<0.001, significantly different from control; one-way ANOVA (Dunnet's *post hoc* test) (C).

MGO is known to induce modification of proteins by glycoxidation [2], [27], [28], [29]. Accumulation of damaged proteins can be toxic and interfere with normal cell function. Therefore, repair or elimination of MGO- or otherwise-damaged proteins is critical to ensure proper cell functioning and survival. This function is supported by a complex quality control mechanism that relies on molecular chaperones and UPS, which can either refold damaged proteins or direct them to proteasomal degradation. The molecular chaperones Hsc70 and Hsp90 bind to unfolded proteins and both contribute to regulate the protein quality control [9], [30], [31], [32]. Therefore, it is likely that destabilization of these chaperones may compromise the protein quality control. Data obtained in this study shows that in addition to inhibiting the proteasome, MGO induces a decrease in the levels of Hsc70 and Hsp90 proteins (Figures 2A, 2B and 2C).

### MGO induces the formation of CHIP-, Hsp40- and ubiquitin-containing aggregates

**Figure 3:**
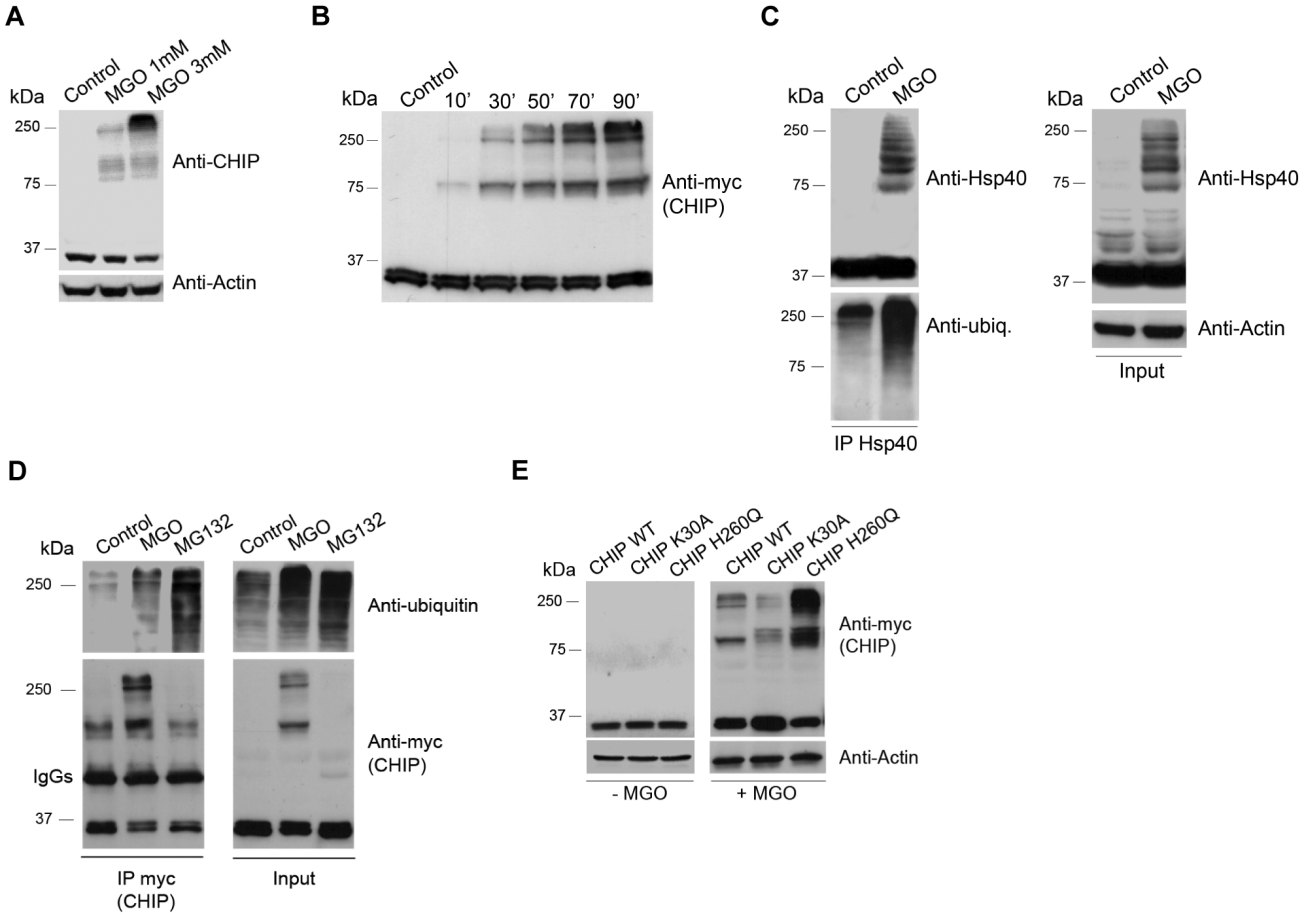
MGO induces formation of large aggregates containing ubiquitin, CHIP and Hsp40. (A) ARPE-19 cells were treated with 1 mM MGO or 3 mM MGO for 3 hours and the cell lysates were analyzed by western blotting using specific antibodies against CHIP and actin. (B) ARPE-19 cells were transfected with myc-CHIP wt and treated with 3 mM MGO for 15, 30, 50, 79 and 90 minutes. Proteins were separated by SDS-PAGE, transferred to PVDF membranes and probed for c-myc. (C) ARPE-19 cells were treated with 1 mM MGO or 3 mM MGO for 3 hours. The cell lysates were used to immunoprecipitate Hsp40 and immunoblot the immunoprecipitates against ubiquitin. (D) ARPE-19 cells were transfected with myc-CHIP wt and treated with 3 mM MGO for 3 hours. c-myc (CHIP) was immunoprecipitated and the immunoprecipitates were probed using a monoclonal antibody against ubiquitin. (E) ARPE-19 cells were transfected with c-myc CHIP wt, c-myc CHIP K30A or c-myc CHIP H260Q. Cells were subsequently treated with 3 mM MGO for 3 hours and the cell lysates were immunoblotted for c-myc and actin.

Hsp40 and CHIP are crucial regulators of the protein quality control [30], [31], [33]. Damaged proteins are often recognized and bind to Hsp40, which in turn recruit Hsc70. Hsp40 functions in cooperation with the co-chaperone CHIP, which has ubiquitin ligase activity and, therefore, is able to ubiquitinate damaged proteins, promoting its UPS-dependent degradation [9]. Data represented in Figures 3A, 3B and 3C show that MGO strongly increases, in a time- and dose-dependent manner, the accumulation of bands with high molecular weights that present immunoreactivity for CHIP and Hsp40 antibodies. Moreover, we observed that MGO increases the ubiquitination of CHIP- and Hsp40-positive aggregates (Figures 3C and 3D). However, ubiquitination on its own does not fully explain the posttranslational modification of CHIP-containing aggregates, since MG132 strongly increases ubiquitination of the protein and does not induce accumulation of bands with high molecular weights, consistent with CHIP-containing aggregates. We suggest that these high molecular weight bands correspond to ubiquitinated substrates that undergo MGO-induced post-translational modifications, ending up in large aggregates containing ubiquitinated proteins, chaperone and co-chaperone complexes, including Hsp40 and CHIP. This could be the result of the cooperative action of chaperones and UPS in rescuing or degrading misfolded and/or post-translationally modified substrates. It is conceivable that some of these aggregates will became insoluble, accounting for age- and diabetes-related cell damage. To further pursue this issue, ARPE-19 cells were transfected with a mutant CHIP that cannot bind chaperones (CHIP K30A) or with a mutant CHIP with no ubiquitin ligase activity (CHIP H260Q). Data shows that the accumulation of bands with high molecular weight, with reactivity for CHIP, decreases when CHIP does not contain chaperone-binding affinity (Figure 3E). Conversely, accumulation of high molecular weight complexes is potentiated by overexpression of CHIP with no ubiquitin ligase activity (Figure 3E). This suggests that the accumulation of CHIP-containing high molecular weight complexes is dependent on the two domains of the protein (ubiquitin-ligase and chaperone-binding) and is likely to be the result of CHIP being trapped in ubiquitinated complexes containing molecular chaperones and damaged/misfolded proteins.

### MGO leads to accumulation of modified proteins and decreases the cell viability

**Figure 4:**
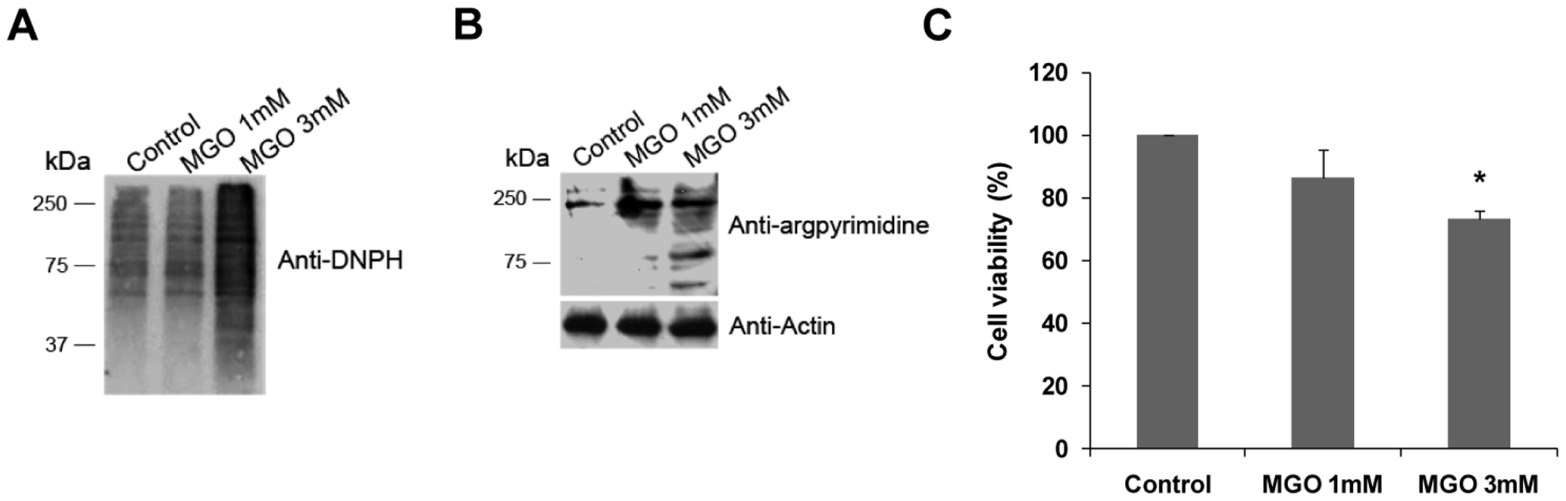
MGO induces accumulation of oxidized and argpyrimidine-modified proteins and decreases cell viability. (A) ARPE-19 cells were treated with 1 mM MGO or 3 mM MGO for 3 hours and the cell lysates were used to derivatize proteins with DNPH. Derivatized proteins were separated by SDS-PAGE, transferred to PVDF membranes and probed with an antibody against dinitrophenylhydrazone derivatives. (B) ARPE-19 cells were treated with 1 mM or 3 mM MGO for 3 hours and the cell lysates were immunoblotted for argpyrimidine and actin. (C) ARPE-19 cell were treated with 1 mM MGO or 3 mM MGO for 3 hours and used to assess cell viability through the MTT colorimetric assay. The quantified results represent the mean ± SD of at least three independent experiments. * p<0.05, significantly different from control; one-way ANOVA (Dunnet's *post hoc* test).

Considering that MGO impairs the ubiquitin-proteasome system and disrupts the chaperone axis, we suggest that MGO is likely to disrupt the protein quality control system in the cell, leading to a potentially toxic accumulation of damaged proteins. Indeed, we observed that MGO strongly increases the levels of oxidized proteins (Figure 4A). Consistently, we also observed increased accumulation of AGEs in the presence of MGO, as revealed by increased immunoreactivity of whole protein extracts against an anti-argpyrimidine antibody (Figure 4B). Of significance, accumulation of damaged and toxic proteins is accompanied by decreased cell viability (Figure 4C).

### MGO elicits a heat-shock response and activates Hsf-1

Stimuli that induce cell stress and protein damage normally activate sensing mechanisms and various forms of cell response to stress, which often include a dramatic change in the pattern of gene expression and increased synthesis of molecular chaperones. Increased expression of heat-shock proteins generally results in repair of modified proteins, promoting cell survival [34]. The inducible expression of HSPs is mainly regulated by the heat shock transcription factor-1 (Hsf-1), that induces transcription of various heat shock genes.

As previously shown, MGO constitutes a potent stress-inducer by impairing UPS activity, leading to the accumulation of damaged and modified proteins in the cell. On the other hand, Hsf-1 is a crucial transcription factor activated under conditions of stress, including accumulation of damaged proteins. Thus, we assessed whether activation of Hsf-1 is part of the cell response to the noxious effects induced by MGO.

**Figure 5:**
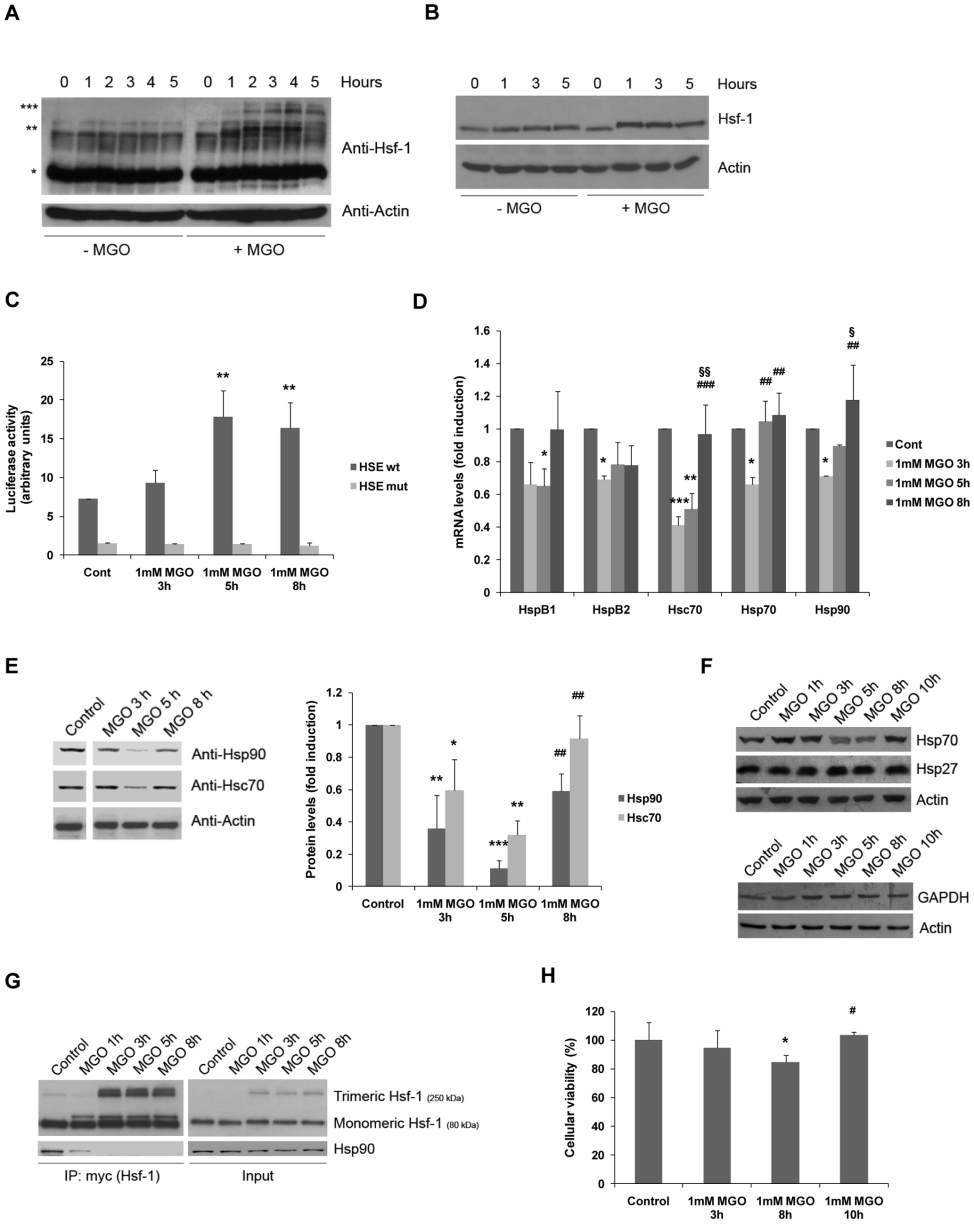
MGO-induced stress activates Hsf-1 through increased oligomerization and DNA-binding, eliciting a cell response to stress. (A) ARPE-19 cells were treated with 50 µM CHX together with 1 mM MGO for 1, 2, 3, 4 or 5 hours and the cell lysates were analyzed by western blot using specific antibodies against Hsf-1 and actin. * Monomeric Hsf-1 (∼80 kDa); ** Dimeric Hsf-1 (∼160 kDa); *** Trimeric Hsf-1 (∼230 kDa). (B) ARPE-19 cells were treated with 1 mM MGO for 1, 3 or 5 hours and the cell lysates were analyzed by western blot using specific antibodies against Hsf-1 and actin. (C) ARPE-19 cells were transiently transfected with the HSE wt (x4)-luciferase or HSE mut (x4)-luciferase vectors and were treated with 1 mM MGO for 3, 5 or 8 hours. Subsequently, the luciferase activity was determined by luminescence. The quantified results represent the mean ± SD of at least three independent experiments. ** p<0.01, significantly different from control; one-way ANOVA (Dunnet's *post hoc* test). (D) ARPE-19 cells were treated with 1 mM MGO for 3, 5 or 8 hours. Total RNA was used to synthesize cDNA, which, in turn, was used as template to quantify HspB1, HspB2, Hsc70, Hsp70 and Hsp90 mRNA and 18S rRNA through RT-PCR. The quantified results represent the mean ± SD of at least three independent experiments. * p<0.05, ** p<0.01, *** p<0.001, significantly different from the respective control; ## p<0.01 and ### p<0.001, significantly different from the corresponding “MGO 3 h” condition; § p<0.05, §§ p<0.01, significantly different from the corresponding “MGO 5 h” condition; one-way ANOVA (Tukey's *post hoc* test). (E) ARPE-19 cells were treated with 1 mM MGO for 3, 5 or 8 hours and the cell lysates were analyzed by western blot using anti-Hsc70, -Hsp90 and -actin antibodies. The quantified results represent the mean ± SD of at least three independent experiments. * p<0.05, ** p<0.01 and *** p<0.001, significantly different from the respective control; one-way ANOVA (Tukey's *post hoc* test); ## p<0.01, significantly different from the corresponding “MGO 5 h” condition; one-way ANOVA (Tukey's ***post hoc*** test). (F) ARPE-19 cells were treated with 1 mM MGO for 1, 3, 5, 8 or 10 hours and the cell lysates were analyzed by western blot using anti-Hsp27, -Hsp70, -GAPDH and -actin antibodies. (G) ARPE-19 cells, transfected with c-myc tagged Hsf-1, were treated with 1 mM MGO for 1, 3, 5 or 8 hours. c-myc was immunoprecipitated and immunoprecipitates were probed against c-myc and Hsp90. (H) ARPE-19 cells were treated with 1 mM MGO for 3, 8 or 10 hours and used to assess cell viability through the MTT colorimetric assay. The quantified results represent the mean ± SD of at least three independent experiments. * p<0.05, significantly different from control; # p<0.05, significantly different from “1 mM MGO 8 h” condition; one-way ANOVA (Dunnet's *post hoc* test).

Oligomerization and phosphorylation of Hsf-1, as well as gain of DNA binding activity, are all well described hallmarks for activation of Hsf-1 [35], [36]. Indeed, in this study we show that Hsf-1 is mostly present in the monomeric/inactive form under normal conditions. However, MGO induces the formation of Hsf-1 homodimers, followed by the formation of Hsf-1 homotrimers (Figure 5A), suggesting Hsf-1 activation. It should be noted that these polymeric forms of Hsf-1 are preserved during the immunoblot procedure due to the low content (1–2%) of β-mercaptoethanol on the denaturation buffer. Consistently, a slight shift on the molecular weight of Hsf-1 is also observed in the presence of MGO (Figure 5B), which may be due to increased phosphorylation under these conditions. To further evaluate the activation of Hsf-1 by MGO-induced stress, we examined the ability of Hsf-1 to bind heat-shock elements (HSE) on DNA and activate transcription of the genes under the control of this conserved sequence. Indeed, through an HSE-luciferase gene reporter assay, we observed that MGO specifically increases the transcriptional activity of Hsf-1 (Figure 5C). Moreover, an increase in the levels of mRNA and protein of the molecular chaperones Hsc70, Hsp70 and Hsp90 further confirm the activation of Hsf-1 by MGO, indicating the activation of a heat-shock response following the exposure to MGO. Interestingly, MGO appears to have a biphasic effect on the regulation of molecular chaperones. In an early stage, MGO decreases the mRNA and protein levels of these proteins (up to 5 h of incubation). However, this response is reverted for longer periods of incubation with MGO (Figure 5D, 5E, 5F), suggesting the mounting of a cell response to stress, which is likely to be triggered by Hsf-1. This response seems to be somewhat specific as we were not able to detect increased levels of Hsp27 (another molecular chaperone whose expression is under the control of Hsf-1) and GAPDH (another cytoplasmic protein) (Figure 5F).

Hsp90-containing multichaperone complexes appear to be the most relevant repressors of Hsf-1 activity [37]. Indeed, in this work we show that oligomerization of Hsf-1 is accompanied by a dramatic decrease in the interaction between Hsp90 and Hsf-1 (Figure 5G), highlighting the critical activation of Hsf-1 in response to MGO.

Of great biological significance, we further noted that activation of Hsf-1 and increased expression of molecular chaperones is accompanied by an increase in cell viability, which is likely to indicate adaptation to the noxious effects of MGO (Figure 5H).

## Discussion

Increased production of MGO, increased modification of proteins by glycoxidation and accumulation of damaged proteins are all hallmarks of aging and of a number of diseases, such as diabetes and cancer [1], [5]. MGO is known to be toxic and to interfere with a plethora of critical mechanisms in the cell. For example, MGO impairs the mitochondria-respiratory chain and increases the production of reactive oxygen species, particularly the anion superoxide, contributing to increased oxidation of proteins [38], [39], [40]. On the other hand, MGO reacts with free ε-NH_2_ groups of basic amino acid residues, through glycation, altering the structure and function of proteins [41]. These and other posttranslational modifications induce exposure of hydrophobic patches of the proteins and loss of secondary and tertiary structure [41], [42]. These changes were proposed to act as a recognition signal for degradation by the proteasome [43], [44], [45]. Canonically, the recognition of the exposed hydrophobic domains of modified proteins is mediated by molecular chaperones, such as Hsc70 and Hsp90. Binding of chaperones is often suggested to recruit the ubiquitin ligase CHIP, which ubiquitinates the modified proteins and targets them for proteasomal degradation [9]. However, the function of the proteasome has also been reported to be impaired upon aging, which may account for inefficient removal of damaged proteins [41], [46], [47]. For example, oxidative inactivation of the proteasome was reported in many types of cells and proteasome activity was shown to decrease in glyoxal-treated cells as compared to controls [41]. Moreover, hyperglycaemia-induced formation of MGO was shown to covalently modify the 20S proteasome, decreasing its activity in cultured endothelial cells and extracts of diabetic kidneys [48]. Consistently, in this study we show that MGO impairs the UPS and destabilizes several molecular chaperones that are crucial for the protein quality control. These impairments are likely to contribute for the accumulation of modified proteins and decreased cell viability.

Indeed, a number of independent studies have shown that MGO modifies and destabilizes several molecular chaperones, such as Hsp27 and α-crystallin [19], [20], [49], [50], [51], [52]. Despite some controversial studies, it is becoming increasingly clear that molecular chaperones and co-chaperones constitute a group of proteins that can be critically affected by MGO. Indeed, in this work, we show that Hsp40, Hsc70, Hsp90 and CHIP are all destabilized by MGO. The protein levels of Hsc70 and Hsp90 decrease in the presence of MGO. On the other hand, CHIP and Hsp40 aggregate following treatment with MGO. These findings led us to hypothesize that, in addition to other established toxic effects, MGO acts by disrupting the cell protein quality control system. Indeed, we show that treatment of cells with MGO leads to the formation of Hsp40- and CHIP-containing ubiquitinated aggregates and that the overexpression of a ligase-dead CHIP increases the levels of CHIP-positive aggregates. Moreover, data from overexpression of a mutant CHIP, which does not bind chaperones, suggests that formation of these aggregates is mediated by ancillary chaperones that bind to CHIP. These findings led us to suggest that CHIP is probably trapped in aggregates of modified proteins, which are recognized by the chaperone-binding site of CHIP and are, at least in part, targeted for degradation mediated by the ubiquitin ligase activity of this protein. Data also emphasizes the dual role of CHIP in the recognition of damaged proteins and in the targeting of these proteins for degradation. Nevertheless, overexpression of CHIP is not sufficient to entirely compensate for the accumulation of modified proteins, thus accounting for increased cell toxicity. Indeed, we show that impairment of UPS and destabilization of molecular chaperones is accompanied by decreased cell viability. Altogether, data is consistent with a model in which MGO-induced post-translational modifications of proteins leads to recruitment of molecular chaperones, in an attempt to repair the misfolded proteins. CHIP is subsequently recruited and acts as a switch, directing substrates from protein refolding to protein degradation. However, our observations suggest that formation of CHIP- and chaperones-containing large ubiquitinated aggregates may become difficult to process by either pathway (refolding or degradation), particularly if the proteasome is also inhibited, leading to the sequestering of CHIP and molecular chaperones in such insoluble protein aggregates.

In agreement with data presented in this work, high levels of MGO and the resulting accumulation of protein aggregates have been extensively correlated with the loss of cell viability and increased cell death [25], [53], [54]. Hsf-1 has been identified as a key player in the cell response to stress by mediating stress-induced heat shock gene expression, by virtue of its ability to bind to DNA, oligomerization and nuclear localization in response to environmental stressors [34], [36]. Indeed, this study shows that Hsf-1 is activated in the presence of increased levels of MGO, as revealed by increased trimerization and increased transcriptional activity. Ultimately, the stress induced by MGO appears to be, at least in part, compensated by the activation of Hsf-1. Activation of Hsf-1 results in overexpression of several heat shock proteins, known to improve refolding of damaged proteins and to promote cell survival. This response suggests that cells have finely tuned sensing mechanisms that counteract abnormal changes induced by stress-inducers. However, these mechanisms are likely to be compromised under acute and severe forms of stress or under chronic mild stresses. Indeed, several reports showed that aging and progression of diabetes are both associated to malfunction and failure of the Hsf-1 system [55], [56], suggesting loss of cell ability to respond to stress and increased susceptibility to cell damage in later stages of age-related diseases.

Regulation of Hsf-1 in the presence of MGO is still unclear but is likely to involve thiol-disulfide exchange, which has been reported to be a redox-dependent posttranslational modification mechanism that can regulate the activity and structure of transcription factors and other proteins [57]. Indeed, NF-κB [58] and AP-1 [59] were shown to be regulated by a mechanism that involves formation of nonnative disulfide crosslinks and oxidation of conserved redox-reactive Cys residues. A similar mechanism is also likely to be involved in regulation of Hsf-1. For example, it was shown that Hsf-1 directly senses both heat and hydrogen peroxide to assemble into a homotrimer in a reversible and redox-regulated manner [35]. The sensing of both conditions requires two Cys residues within the Hsf-1 DNA-binding domain that are engaged in redox-sensitive disulfide bonds. Moreover, Hsf-1 derivatives in which the Cys residues were mutated are refractive to stress-inducible trimerization and DNA binding [60]. In further support of this hypothesis, MGO is known to change the cellular redox status [61], induce the formation of disulfide-crosslinks and modify the SH-group of Cys residues [62], [63]. These evidences may explain the higher preservation of Hsf-1 homodimers and homotrimers when samples are lysed using low amounts of β-mercaptoetanol.

Another interesting aspect that is suggested by the current work is that MGO targets lysine residues, which are major ubiquitin-acceptor sites. At a first glance it might be expected a general decrease on the total levels of ubiquitinated proteins in the cells. However, in this study we observe a clear increase in the levels of ubiquitinated proteins following treatment with MGO. This apparent contradiction is not without precedent. Indeed, there are several reports showing that modification of lysine residues by processes such as lysine acetylation may either increase or decrease ubiquitination, depending on the substrate (reviewed in [64]). In the case of glycation, it is known that CML-modified proteins are substrates for ubiquitin conjugation [41] and that, for example, Raf-1 is degraded by an ubiquitin-proteasome-dependent mechanism in response to MGO [65], suggesting increased ubiquitination of glycated proteins and the existence of a crosstalk between both processes. Considering the data presented in this study, as well as other reports in the literature, it is likely that glycation on lysine residues can direct substrates to either refolding or proteasome degradation. The final fate of a specific substrate is probably the result of the stability of the modified protein, as well as of the recruitment of a number of different binding partners that may divert substrates to specific pathways. However, this is a matter for further investigation.

As a major conclusion, the data obtained in this work suggests that accumulation of MGO, which occurs in a variety of situations, such as diabetes and aging, impairs both the UPS and the protein quality control dependent on CHIP and molecular chaperones, leading to accumulation of toxic aggregates and decreased cell viability. However, these MGO induced changes appear to elicit a response from the Hsf-1 system, which is crucial to help cells to cope with cellular stress and to re-establish homeostasis.

## Materials and Methods

### Cells Culture and Treatments

The human retinal pigment epithelium cell line ARPE-19 (LGC Promochem, Teddington, UK) was cultured in Ham's F12/Dulbecco's modified Eagle's medium (DMEM) (1∶1) supplemented with 10% fetal bovine serum (FBS), antibiotics (100 U/ml penicillin, 100 µg/ml streptomycin and 250 ng/ml amphotericin B) and GlutaMax (1x). All media, GlutaMax and non-essential amino acids were purchased from Invitrogen (Carlsbad, CA, USA). When appropriated, cells were treated with cycloheximide (CHX; Sigma-Aldrich, St. Louis, MO, USA), methylglyoxal (MGO, Sigma-Aldrich, St. Louis, MO, USA) and/or MG132 (Z-LLL-CHO, Calbiochem, San Diego, CA, USA).

### Western Blot Analysis

After appropriate treatments, cells were washed twice in phosphate-buffered saline (PBS), denatured with 2× Laemmli buffer, boiled at 100°C and sonicated. Whole cell extracts were resolved by SDS-PAGE and electrophoretically transferred onto polyvinylidene fluoride (PVDF) membranes. The membranes were blocked with 5% nonfat milk in TBS-T (20 mM Tris, 150 mM NaCl, Tween 0.2%, pH 7.6) and probed for several proteins. The following antibodies were used: mouse anti-actin clone C4 1∶1,000 (Millipore-Chemicon, Billerica, MA, USA), mouse anti-ubiquitin clone P4D1 1∶1,000 (Covance, Princeton, NJ, USA), mouse anti-c-myc clone 9E10 1∶500 (Zymed-Invitrogen, Carlsbad, CA, USA), mouse anti-argpyrimidine 1∶500 (kindly provided by Dr. Koji Uchida from Nagoya University) [20], mouse anti- Hsp70 clone C92F3A-5 1∶1,000 (Stressgen-Enzo Life Sciences, Farmingdale, NY, USA), rat anti-Hsp90 clone 16F1 1∶1,000 (Stressgen-Enzo Life Sciences, Farmingdale, NY, USA), rat anti-Hsc70 clone 1B5 1∶2,000 (Stressgen-Enzo Life Sciences, Farmingdale, NY, USA), rat anti-Hsf-1 clone 10H8 1∶1,000 (Stressgen-Enzo Life Sciences, Farmingdale, NY, USA), rabbit anti-Hsp40 1∶2,000 (Stressgen-Enzo Life Sciences, Farmingdale, NY, USA), rabbit anti-Hsp27 1∶20,000 (Stressgen-Enzo Life Sciences, Farmingdale, NY, USA), rabbit anti-GAPDH 1∶5000 (Cell Signaling, Beverly, MA, USA), goat anti-STUB1/CHIP 1∶500 (Abcam, Cambridge, UK) and horseradish peroxidase-conjugated secondary goat anti-mouse, goat anti-rat, goat anti-rabbit and rabbit anti-goat (Bio-Rad, Hercules, CA, USA). Immunoreactive bands were visualized with an ECL system (GE Healthcare Bio-Sciences, Uppsala, Sweden).

### Determination of Intracellular Concentration of Methylglyoxal

ARPE-19 cells cultured in 60×15 mm plates were lysed with 200 µl of lysis buffer (50 mM Tris-HCl pH 7.4, 250 mM NaCl and 1× protease inhibitor cocktail), incubated for 1 hour on ice and briefly sonicated. Protein concentration was determined by the Coomassie Protein Assay (Pierce-Thermo Scientific, Waltham, MA, USA). Subsequently, 100 µl of lysate (at a concentration of 1 µg/µl) was mixed with 100 µl HCl (0.2 M) and the intracellular concentration of MGO was determined based on a previously reported method [66]. Briefly, 50 µl of sample was mixed with 200 µl of 0.1 M potassium phosphate buffer pH 7.0, 200 µl of ethanol and 50 µl of freshly prepared 20 mM DDB (dissolved in 10 mM HCl) (Invitrogen, Carlsbad, CA, USA). The mixture was incubated at room temperature for at least four hours and then centrifuged during 10 minutes at 16,000 g. Subsequently, MGO was determined through reverse-phase HPLC. The HPLC system consisted of a L-6200A Intelligent Pump, a F-1080 Fluorescence Detector (Hitachi, Tokyo, Japan) and a Waters µBondapak™ C18 column (10 µM, 3.9×300 mm) (Waters, Milford, MA, USA). Mobile phase A was a mixture of 10 mM potassium phosphate buffer (pH 3.0) and acetonitrile (90/10, v/v). Mobile phase B consisted of a mixture of acetonitrile and water (70/30, v/v). Samples (20 µl) were injected and separation was performed with a linear gradient from 0–100% mobile phase B over 28 minutes. The flow rate was set at 1.0 ml/min and fluorescence detection was performed with excitation and emission wavelengths of 352 nm and 385 nm, respectively. Levels of MGO were determined by integration of peak areas using appropriate external standards.

### Measurement of the 20S Proteasome Activity

Cells were washed twice with PBS, lysed with a Tris buffer (50 mM Tris pH 7.4, 1 mM DTT) and sonicated. After centrifugation (16,000 g for 10 minutes at 4°C), protein concentration was determined using the Coomassie method and 40 µg of protein was incubated with the following fluorogenic substrates: 100 µM Suc-LLVY-MCA for the chymotrypsin-like activity (Biomol-Enzo Life Sciences, Farmingdale, NY, USA); 25 µM Boc-LRR-MCA for the trypsin-like activity (Biomol-Enzo Life Sciences, Farmingdale, NY, USA); 150 µM Z-LLE-MCA for the caspase-like activity (Calbiochem, San Diego, CA, USA). The proteasome activities were monitored during 1 hour at 37°C, in periods of 5 minutes (excitation wavelength at 380 nm; emission wavelength at 460 nm). Absorbance was measured on a Biotek Synergy HT spectrophotometer (Biotek, Winooski, VT, USA), using the Gen 5 software to monitor the results (Biotek, Winooski, VT, USA).

### Transient Transfection

One day before transfection, cells were seeded in 60×15 mm plates in 2.5 ml of medium so that the cells were 90% confluent at the time of transfection. 4 µg of plasmid DNA were used per dish and transfections were carried out using Lipofectamine 2000 (Invitrogen, Carlsbad, CA, USA), according to the manufacturer's specifications. The following plasmids were used: pcDNA3.1 c-myc-CHIP wt, pcDNA3.1 c-myc-CHIP K30A, pcDNA3.1 c-myc-CHIP H260Q (originally provided by Dr. C. Patterson from the North Carolina University) [67]; pcDNA3.1 hHsf-1-c-myc (provided by Dr. Lea Sistonen from the University of Turku, Turku, Finland) [68]; pGL4.23 HSE wt (x4)-Luciferase, pGL4.23 HSE mut (x4)-Luciferase (provided by Dr. Ueli Schibler from the University of Geneva, Switzerland) [69].

### Luciferase-Reporter Assay

Subconfluent ARPE-19 cells, plated in 24-well plates, were transfected with pGL4.23 HSE wt (x4)-Luciferase or pGL4.23 HSE mut (x4)-Luciferase [69], using Lipofectamine 2000 (Invitrogen, Carlsbad, CA, USA). The plasmids pGL4.23 HSE-Luciferase contain four copies of the wild-type or mutated form of a heat shock element (HSE) and a minimal promoter upstream of a firefly luciferase reporter gene [69]. The mutant form does not bind the transcription factor Hsf-1. Twenty-four hours after transfection, cells were treated as mentioned and then assayed for luciferase activity, which was measured using a LMax II 384 ROM v1.04 reader and SoftMax Pro 5 software (Molecular Devices, Sunnyvale, CA, USA), as described by the manufacturer's protocol.

### Real-Time PCR

Following the relevant treatments, total RNA was purified according to the manufacturer's specifications of Qiagen RNeasy mini kit (Qiagen, Valencia, CA, USA) and treated with RNase-free DNase I (GE Healthcare Bio-Sciences, Uppsala, Sweden). SuperScript II Reverse Transcriptase (Invitrogen, Carlsbad, CA, USA) and random hexadeoxynucleotide primers were used to synthesize cDNA. For the cDNA real-time PCR, it was used the SYBR Green PCR master mix (Bio-Rad, Hercules, CA, USA), according to the manufacturer's instructions, and the cDNA amplification was performed using the following sets of primers: hHsc70 forward 5′-ATACCTCCTGCACCCCGAG-3′; hHsc70 reverse 5′-TTGCTCAAACGGCCCTTGTC-3′; hHsp70 forward 5′-GTGCAGTTG CCTACAGGATTAAC-3′; hHsp70 reverse 5′-TCGGCTGTCTCCTTCAGTTTG-3′; hHsp90 forward 5′-TTCCACGTCTCTGCATTCC-3′; hHsp90 reverse 5′-CTTGGGTCTG GGTTTCCTC-3′; hHspB1 forward 5′-GCTGACGGTCAAGACCAAGG-3′; hHspB1 reverse 5′-GGGGGCAGCGTGTATTTCC-3′; hHspB2 forward 5′-C GAGTACGAATTT GCCAACC-3′; hHspB2 reverse 5′-AGTAGCCATGGTAGAGTGTG-3′; 18S rRNA forward 5′-GTCTGCCCTATCAACTTTC-3′; 18S rRNA reverse 5′-TTCCTTGGATGTG GTAGC-3′ (endogenous control). The real time PCR analyses were conducted on a ABI Prism 7000 quantitative PCR system (Applied Biosystems, Foster City, CA, USA).

### Immunoprecipitation

Cells cultured in 60×15 mm plates were washed twice with PBS, scraped off the dishes and collected in ice-cold PBS. Pellets were resuspended in 100 µl of 50 mM Tris-HCl pH 7.4, 150 mM NaCl, 10 mM IOD, 2 mM PMSF, 20 mM Na_3_MoO_4_, 0.25% NP-40 and protease inhibitor cocktail (Roche Applied Science, Indianapolis, IN, USA) and incubated for 30 minutes on ice. Following centrifugation at 16,000 g for 10 minutes, supernatants were transferred to new tubes, 2.5 µg of anti-c-myc or anti-Hsp40 were added and incubated overnight at 4°C with gentle agitation. Thereafter, 50 µl of protein G-Sepharose (GE Healthcare Bio-Sciences, Uppsala, Sweden) were added and incubations proceeded at 4°C for 2 hours. Beads were washed 3 times and the immunoprecipitated proteins were eluted with 2× Laemmli buffer and boiled at 100°C. Eluted samples were used to perform western blot analyses.

### Protein derivatization with DNPH

After the treatments, ARPE-19 cells were washed twice with ice-cold PBS and lysed in Tris-HCl pH 7.6 supplemented with 1% NP-40 and protease inhibitor cocktail (Roche Applied Science, Indianapolis, IN, USA). For derivatization of carbonyl-containing proteins, equal amounts of proteins were mixed with equal volume of 10 mM 2,4-dinitrophenylhydrazine (DNPH) in 10% trifluoroacetic acid and incubated at room temperature for 15 minutes. The reaction was stopped by precipitation of proteins with 20% trichloroacetic acid (TCA). The pellet was washed with ethylacetate:ethanol (1∶1) to remove free DNPH. Subsequently, the pellet was solubilized with Laemmli buffer. Samples were resolved by SDS-polyacrylamide gel electrophoresis (SDS-PAGE) and transferred to PVDF membranes. The membrane was probed with antibody to dinitrophenylhydrazone derivatives (Dako, Glostrup, Denmark).

### MTT cell viability assay

After the treatments, ARPE-19 cells seeded onto 24-well plates were washed twice with PBS and incubated with 0.5 mg/ml MTT [3-(4,5-dimethylthiazol-2-yl)-2,5-diphenyltetrazolium bromide; Invitrogen, Carlsbad, CA, USA], solubilized in Krebs buffer (130 mM NaCl, 4 mM KCl, 1.5 mM MgCl_2_, 1 mM CaCl_2_, 6 mM glucose, 10 mM HEPES, pH 7.4), for 2 hours at 37°C in a cell culture incubator. Subsequently, supernatants were removed and the precipitated dye was dissolved in 300 µl 0.04 M HCl (in isopropanol) and colorimetric quantified at a wavelength of 570 nm, with wavelength correction at 620 nm, using a Biotek Synergy HT spectrophotometer (Biotek, Winooski, VT, USA).

### Statistical Analysis

Data are reported as the means ± standard deviation (SD) of at least three independent experiments. Comparison between multiple groups was performed by one-way analysis of variance test (ANOVA) using GraphPad Prism 5.0 software (GraphPad Software). For comparison between two groups, the paired *t* test was used. In all cases, *p<*0.05 was considered significant.
